# Effects of turbulence on diatoms of the genus *Pseudo-nitzschia* spp. and associated bacteria

**DOI:** 10.1093/femsec/fiae094

**Published:** 2024-07-10

**Authors:** Yanis Maire, François G Schmitt, Konstantinos Kormas, Sotirios Vasileiadis, Amandine Caruana, Dimitra-Ioli Skouroliakou, Vasileios Bampouris, Lucie Courcot, Fabienne Hervé, Muriel Crouvoisier, Urania Christaki

**Affiliations:** Université du Littoral Côte d'Opale, CNRS, Université de Lille, UMR 8187 LOG, 32 Ave. Foch, F-62930 Wimereux, France; Université du Littoral Côte d'Opale, CNRS, Université de Lille, UMR 8187 LOG, 32 Ave. Foch, F-62930 Wimereux, France; Department of Ichthyology and Aquatic Environment, School of Agricultural Sciences, Fitoko st. 1, 38446 Volos, Greece; Agricultural Development Institute, University Research and Innovation Centre “IASON”, Argonafton & Filellinon, 38221, Greece; Agricultural Development Institute, University Research and Innovation Centre “IASON”, Argonafton & Filellinon, 38221, Greece; Department of Biochemistry and Biotechnology, Viopolis 41500, University of Thessaly, Larissa, Greece; IFREMER, PHYTOX, Laboratoire PHYSALG, BP21105, Rue de l'Ile d'Yeu, F-44300 Nantes, France; Université du Littoral Côte d'Opale, CNRS, Université de Lille, UMR 8187 LOG, 32 Ave. Foch, F-62930 Wimereux, France; Université du Littoral Côte d'Opale, CNRS, Université de Lille, UMR 8187 LOG, 32 Ave. Foch, F-62930 Wimereux, France; Department of Ichthyology and Aquatic Environment, School of Agricultural Sciences, Fitoko st. 1, 38446 Volos, Greece; Université du Littoral Côte d'Opale, CNRS, Université de Lille, UMR 8187 LOG, 32 Ave. Foch, F-62930 Wimereux, France; IFREMER, PHYTOX, Laboratoire PHYSALG, BP21105, Rue de l'Ile d'Yeu, F-44300 Nantes, France; Université du Littoral Côte d'Opale, CNRS, Université de Lille, UMR 8187 LOG, 32 Ave. Foch, F-62930 Wimereux, France; Université du Littoral Côte d'Opale, CNRS, Université de Lille, UMR 8187 LOG, 32 Ave. Foch, F-62930 Wimereux, France

**Keywords:** bacteria, domoic acid, *Pseudo-nitzschiafraudulenta*, *Pseudo-nitzschia multiseries*, turbulence

## Abstract

Turbulence is one of the least investigated environmental factors impacting the ecophysiology of phytoplankton, both at the community and individual species level. Here, we investigated, for the first time, the effect of a turbulence gradient (Reynolds number, from Re_λ_ = 0 to Re_λ_ = 360) on two species of the marine diatom *Pseudo-nitzschia* and their associated bacterial communities under laboratory conditions. Cell abundance, domoic acid (DA) production, chain formation, and Chl *a* content of *P. fraudulenta* and *P. multiseries* were higher for intermediate turbulence (Re_λ_ = 160 or 240). DA was detectable only in *P. multiseries* samples. These observations were supported by transcriptomic analyses results, which suggested the turbulence related induction of the expression of the DA production locus, with a linkage to an increased photosynthetic activity of the total metatranscriptome. This study also highlighted a higher richness of the bacterial community associated with the nontoxic strain of *P. fraudulenta* in comparison to the toxic strain of *P. multiseries. Bacillus* was an important genus in *P. multiseries* cultures (relative abundance 15.5%) and its highest abundances coincided with the highest DA levels. However, associated bacterial communities of both *Pseudo-nitzschia* species did not show clear patterns relative to turbulence intensity.

## Teaser

The effect of a turbulence gradient—from ‘still’ to ‘storm’ conditions—was investigated, for the first time, on two species of potentially toxic diatom (*Pseudo-nitzschia*) and their associated bacterial communities under laboratory conditions.

## Introduction

Turbulence in the oceans, generated by various factors like winds, cooling, evaporation, tides (Thorpe 2005), and waves, has a profound impact on marine phytoplankton (e.g. Estrada and Berdalet 1997, Schmitt 2020). In three-dimensional turbulence, there is a cascade of eddies from large to small scales, until the Kolmogorov scale where viscous dissipation becomes dominant, which is of the order of 1000 µm in the ocean’s surface waters. Passive scalars, such as temperature, salinity, or the concentration in nutrients, are transported by turbulence; their fluctuations are generated at large scales and transported through successive breakdowns into smaller scales until the Batchelor scale, where the effect of molecular diffusion become important in comparison to turbulent mixing (Batchelor 1959). The Batchelor scale is of the order of 10–100 µm in the ocean’s surface waters. Diatoms, ranging from 2 to 200 µm, have a size similar to the Batchelor scale and are much smaller than the Kolmogorov scale. This means that these phytoplankton cells experience laminar shears due to turbulence (Estrada and Berdalet 1997, Peters and Marrasé 2000) and that turbulence may have effects on phytoplankton cells by increasing the diffusion mixing of nutrients to the cell surfaces (Estrada and Berdalet 1997, Sullivan et al. 2003). Phytoplankton ecologists have long observed that turbulence levels in the water column impact phytoplankton communities in relation to the shape, size, and swimming capacity of phytoplankton species. Published 45 years ago, a concept known as ‘Margalef’s Mandala’, categorized phytoplankton into groups based on nutrient availability and turbulence intensity. It showed that diatoms are favoured by highly turbulent and nutrient-rich conditions, while dinoflagellates preferred the opposite conditions (Margalef 1978). This conceptualization has been verified in several marine systems and is now generally accepted in marine phytoplankton ecology. Yet, there is also substantial evidence indicating that turbulence impacts various factors crucial for diatom cell survival including: nutrient availability (Estrada and Berdalet 1997, Pahlow et al. 1997, Dell’Aquila et al. 2017), settling velocity (Estrada and Berdalet 1997, Ruiz et al. 2004), chain structure (Clarson et al. 2009, Amato et al. 2017, Dell’Aquila et al. 2017), gene expression (Amato et al. 2017), and interactions with grazers and diatom–diatom encounter rates related to reproduction and chain formation (Rothschild and Osborn 1988).

In spite of its potential impact, little is known about the effect of turbulence on the diatom *Pseudo-nitzschia* spp. This is a critical gap given that *Pseudo-nitzschia* spp. can form harmful algal blooms (Bates et al. 2018) and climate change is anticipated to modify turbulence in the oceans via two opposite processes: increase in the water column stratification due to the warming of the upper ocean, and increase in the frequency and intensity of extreme climatic events such as storms (IPCC 2023). There is, therefore, a need to evaluate how and whether turbulence can influence *Pseudo-nitzschia* spp.’s growth and toxicity.

The factors triggering the blooms and toxicity [via the production of domoic acid (DA)] of *Pseudo-nitzschia* spp. are not completely resolved but temperature, salinity, nutrients, irradiance, photoperiod, association with bacteria, and upwelling events have been found to influence its growth and toxicity (Bates et al. 1995, Bates 1998, Lelong et al. 2012, Trainer et al. 2012, 2018). Some of these factors can directly or indirectly interact with turbulence (Estrada and Berdalet 1997, Arnott et al. 2021). For example, by influencing the degree of mixing of phytoplankton cells within the water column, turbulence intensity impacts the cells access to the surface photic layer and exposure to light/dark conditions.

The hologenome concept (Zilber-Rosenberg and Rosenberg 2008) refers to all eukaryotic organisms, including unicellular algae. *Pseudo-nizschia* spp. live in association with a microbiota composed of bacteria. Turbulence may also impact the relationships between *Pseudo-nizschia* spp. and their associated bacteria. The bacteria associated to diatoms can be classified into two categories: (i) the free-living bacteria, which are attracted by the organic matter exudated by phytoplankton cells and are motile, ‘swimming’ towards the cell in a zone called the phycosphere; and (ii) the epiphytic bacteria, which are attached to phytoplankton cells. The phytoplankton–bacterial interactions are governed by the production of metabolites by both communities, which can either be beneficial or harmful (e.g. Grossart 1999, Seymour et al. 2017). The bacteria associated with *Pseudo-nitzschia* spp. often belong to different classes such as *Gammaproteobacteria* (mainly represented by the genus *Marinobacter* and *Alteromonas*), *Alphaproteobacteria* (mainly represented by *Phaeobacter*) and *Bacilli* (e.g. Guannel et al. 2011, Lelong et al. 2012, Sison-Mangus et al. 2016). Because a part of the DA produced by toxic *Pseudo-nizschia* strains can be released to the media (Lelong 2012, Trainer et al. 2012), some researchers have hypothesized that the *Pseudo-nitzschia* spp./bacteria relationship may influence the production of DA by *Pseudo-nitzschia* spp. and would modulate the composition of the associated heterotrophic bacterial community (Bates et al. 1995, Guannel et al 2011, Lelong et al. 2012, Sison-Mangus et al 2016). Although, the potential effects of associated bacteria on *Pseudo-nitzschia* spp. growth and production of DA are not resolved; it has been reported that DA production and growth of axenic cultures of *Pseudo-nitzschia* spp. are reduced in comparison to nonaxenic cultures (Lelong et al. 2012 and references therein).

Given all the above, it can be hypothesized that turbulence could influence *Pseudo-nitzschia* spp. and that different levels of turbulence are likely to modify the relationship between *Pseudo-nitzschia* spp. and associated bacteria with potential consequences on the production of DA. To explore this, laboratory experiments were conducted by exposing two species of *Pseudo-nitzschia* (*Pseudo-nitzschia fraudulenta* and *Pseudo-nitzschia multiseries*), cultured under nonaxenic conditions, to five levels of turbulence. The control of turbulence intensity was made with the Agiturb turbulence generation system, designed to generate a precise, quantifiable, and homogenous turbulent flow (Le Quiniou et al. 2022). Cell abundance, chain formation, and toxicity of the two *Pseudo-nitzschia* species were measured, and the composition of the associated epiphytic bacteria was determined by metabarcoding (16S RNA gene amplicon sequencing).

## Materials and methods

### *Pseudo-nitzschia* strains and culture maintenance

Two nonaxenic *Pseudo-nitzschia* strains were used*. Pseudo-nitzschia multiseries* NWFSC713 was isolated from Puget Sound in the USA (provided by V. Trainer, NOAA, Marine Biotoxins Program, USA) and *Pseudo-nitzschia fraudulenta* PNfra 20–6 was isolated in the eastern English Channel (provided by J. Fauchot, UNICAEN, CNRS UMR 8067, BOREA, France). The cultures were grown in K/2 medium (Keller et al. 1987), at 15°C, with a 12L:12D photoperiod and under an irradiance of $100{\mathrm{\ \mu mol\ photon\ }}{{\mathrm{m}}}^{ - 2}{{\mathrm{s}}}^{ - 1}$. The autoclaved K/2 medium was prepared with natural seawater from the eastern English Channel aged in the dark for several months before use. The cultures were grown in order to obtain large volumes (5 l) needed for the experimental requirements. No turbulence was applied in the cultures.

### Agiturb turbulence generation system

The experiments were conducted with the Agiturb turbulence generation system (Le Quiniou et al. 2022). It is based on the ‘Four-roll mill’ system proposed in 1934 by Taylor, which generates a strain-dominated two-dimensional laminar flow using four rolls with contra-rotating rates (Taylor 1934, Wereley and Gui 2003). The Agiturb system has some differences: it is a cubic tank with a maximum capacity of 38 ’l filled with 15 l of medium, under which four agitators are placed. The four agitators are contra-rotating at the same velocity (Ω), which can be changed from 100 r m^−1^ (revolutions per minute) to 900 r m^−1^ (Table 1). Energy is injected into the flow through the motion of four stirring bars (3.8 cm long with a diameter of 0.8 cm) activated by four magnetic stirrers (VELP MST Digital 5 l) situated at symmetric positions (Fig. S1). The energy dissipation rate (ε) in developed turbulent flows (i.e. statistically homogenous and isotropic; Pope 2000) is provided by the following equation:


(1) \begin{eqnarray*}
\epsilon \approx \frac{{{{{\mathrm{\tilde{u}}}}}^3}}{{\mathrm{L}}},
\end{eqnarray*}

where L is the scale at which the energy is injected in the system, and is of the order of the distance between the two adjusted agitators (Fig. S1). In this case, ${\mathrm{L}} = 16.8{\mathrm{\ cm}}$ and ${\mathrm{\tilde{u}}}$ is the mean fluctuating velocity as,


(2) \begin{eqnarray*}
{{\mathrm{\tilde{u}}}}^2 = \frac{2}{3}{\mathrm{K}},
\end{eqnarray*}

with ${\mathrm{K}}$, the kinetic energy. These parameters permit the scale of the smallest eddies to be calculated: ${\mathrm{\eta }}$ the Kolmogorov scale (3),


(3) \begin{eqnarray*}
{\rm \eta} = \left(\frac{{\rm \nu}^3}{\epsilon} \right )^{1/4},
\end{eqnarray*}

where, ${\mathrm{\nu }} = 1.1{\mathrm{\ x\ }}{10}^{ - 6}{\mathrm{\ }}{{\mathrm{m}}}^2{{\mathrm{s}}}^{ - 1}{\mathrm{\ }}$for ${\mathrm{T}} = 18 \pm 2{\mathrm{\ }}^\circ {\mathrm{C}}$, is the kinematic viscosity (Schmitt 2020). The Taylor scale *λ* (4) has no clear physical interpretation but is useful for the comparison between different types of turbulent flow via the Taylor-based Reynolds number (${\mathrm{R}}{{\mathrm{e}}}_{\mathrm{\lambda }}$; 5).


(4) \begin{eqnarray*}
{\mathrm{\lambda }} = \sqrt {15} {{\mathrm{\eta }}}^{2/3}{{\mathrm{L}}}^{1/3}.
\end{eqnarray*}

(5) \begin{eqnarray*}
{\mathrm{R}}{{\mathrm{e}}}_{\mathrm{\lambda }} = \frac{{{\mathrm{\tilde{u}\lambda }}}}{{\mathrm{\nu }}}.
\end{eqnarray*}

**Table 1.** Turbulence intensities used during the experiments.

Rotation (RPM)	Week	${\mathrm{R}}{{\mathrm{e}}}_{\mathrm{\lambda }}$ (Reynolds number)	Dissipation (${{\mathrm{m}}}^2.{{\mathrm{s}}}^{ - 3}$)	Turbulence
0	1 and 2	$0$	$0$	Still condition
100	2	$130$	${3.10}^{ - 6}$	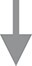
200	1	$160$	${10}^{ - 5}$	
400	1	$240$	${10}^{ - 4}$	
900	2	$360$	${10}^{ - 3}$	Storm

From the above equations and the experimental procedure described by Le Quiniou et al. (2022) the characteristics of the different levels of turbulence were derived. These levels correspond to different conditions in the aquatic environment. Much of the surface open ocean typically exhibits average dissipation rates of the order ${10}^{ - 10}$–${10}^{ - 6}{\mathrm{\ }}{{\mathrm{m}}}^2.{\mathrm{\ }}{{\mathrm{s}}}^{-3}{\mathrm{\ }}$ (Barton et al. 2014). More energetic zones including tidal channels, fronts, storms, and breaking waves, may generate very high dissipation rates of the order of ${10}^{ - 5}$–${10}^{ - 4}{\mathrm{\ }}{{\mathrm{m}}}^2.{{\mathrm{s}}}^{-3}$, while rates of the order of ${10}^{ - 3}\ {{\mathrm{m}}}^2.{\mathrm{\ }}{{\mathrm{s}}}^{-3}$ correspond to storm conditions (Dell’aquila et al. 2017). The dissipation rate applied in our experiments varied from ${10}^{ - 3}{\mathrm{\ }}$–${10}^{ - 6}{{\mathrm{m}}}^2.{\mathrm{\ }}{{\mathrm{s}}}^{-3}$. Zero turbulence condition acted as negative control referred here as ‘still condition’, (Table 1, Fig. S1). The level of turbulence is characterized by the Taylor-based Reynolds number (${\mathrm{R}}{{\mathrm{e}}}_{\mathrm{\lambda }}$), the value of the kinetic energy ${\mathrm{K}}$, and the dissipation rate $\epsilon $. The values of these three parameters as a function of the speed of rotation are known. The Taylor-based Reynolds number referred to as ‘Reynolds number’ (${\mathrm{R}}{{\mathrm{e}}}_{\mathrm{\lambda }}$) will be used hereafter (Table 1).

### Experimental set-up and sampling

The experiments were performed in 38 l tanks (Fig. S1). The five turbulence intensities were applied in triplicate. For each experiment nine Agiturb systems were used. For every strain the experiments lasted 3 days and were held during two consecutive weeks. In the first week (referred to here as W1), the still condition ${{\mathop{\mathrm{Re}}\nolimits} }_\lambda = 0,\,{{\mathop{\mathrm{Re}}\nolimits} }_\lambda = 160$, and ${{\mathop{\mathrm{Re}}\nolimits} }_\lambda = 240$ turbulence levels were applied. In the second week (referred to here as W2), the still condition ${{\mathop{\mathrm{Re}}\nolimits} }_\lambda = 0,\,{{\mathop{\mathrm{Re}}\nolimits} }_\lambda = 130,\,{{\mathop{\mathrm{Re}}\nolimits} }_\lambda = 360$ were applied. The tanks were filled with 15 l of 0.2 µm filtered and autoclaved seawater then K/2 medium were added in each tank so that diatoms were grown in nutrient replete conditions (targeted initial concentrations NaNO_3_ = 28 µM, ${\mathrm{N}}{{\mathrm{a}}}_2{\mathrm{Si}}{{\mathrm{O}}}_3.9{{\mathrm{H}}}_2{\mathrm{O}} = 45\,{\mathrm{\rm \mu M}}$, ${\mathrm{K}}{{\mathrm{H}}}_2{\mathrm{P}}{{\mathrm{O}}}_4 = 18\,{\mathrm{\mathit{\rm\mu} M}}$). The tanks were covered with a transparent glass to minimize contamination from the air. The cell abundance in cultures (which were in exponential growth phase) were checked prior of each experiment, and the culture was added in each tank to an approximate abundance of $1000{\mathrm{\ cells\ m}}{{\mathrm{l}}}^{ - 1}$. This is relatively high initial concentration since *Pseudo-nitzschia* blooms typically reach abundances of medium to high 10^6^ cells l^−1^ (e.g Trainer et al. 2012, Bates et al. 2018, and references therein)

All the experiments were run in a thermoregulated laboratory in the same conditions as the ones used for culture maintenance (i.e. 15°C, with a 12L:12D photoperiod and under an irradiance of $100{\mathrm{\ \mu mol\ photons\ }}{{\mathrm{m}}}^{ - 2}.{{\mathrm{s}}}^{ - 1}$).

The initial culture was maintained in exponential growth phase for the W2 experiment. At each time point (${{\mathrm{T}}}_0,{\mathrm{\ }}{{\mathrm{T}}}_{24},{\mathrm{\ }}{{\mathrm{T}}}_{48},{\mathrm{\ }}$and ${\mathrm{\ }}{{\mathrm{T}}}_{72}\ $ h), 500 ml were sampled with a sterile tube from each tank and immediately subsampled for the measurements of Chl *a*, nutrients, cell abundance and chain formation. Chl *a* concentrations were measured by fluorometry as described by Lorenzen (1966). Inorganic nutrient concentrations, nitrate (NO_3_^−^), nitrite (NO_2_^−^), phosphate (PO_4_^3−^), and orthosilicic acid (Si(OH)_4_) were analyzed according to Aminot and Kérouel, (2004) with a SEAL AA3 HR chemistry analyzer.

Samples (10 ml) were fixed with glutaraldehyde solution (1% v/v). For diatom cell counts, a Nageotte counting chamber using a Zeiss Imager M2 (magnification 100x) was used. The number of cells of the chains was calculated by counting the number of cells in every chain up to a total of 100 cells count. A proxy of growth of diatoms was estimated for each tank and each time point as the ratio of the cell abundance at a given time point to abundance at T0 (e.g. ${{\mathrm{N}}}_{48}/{{\mathrm{N}}}_0$).

Free-living bacterial and viral abundances were monitored by flow cytometry to ensure that the results were not skewed by an exceptional bacterial and/or viral proliferation during our experiments. For free-living heterotrophic bacteria and virus abundance, 2 ml samples were fixed with glutaraldehyde at a final concentration of 1%, stored at 4°C for 40 min, flash frozen in liquid nitrogen, and then kept at −80°C until analysis with a Cytoflex cytometer (Beckman Coulter). Counts for heterotrophic bacteria and virus-like particles (VLP) were made after staining with SYBRGreen based on their green fluorescence (Marie et al. 1999, Brussaard 2004, respectively). Two heterotrophic bacterial populations were discriminated, one with high fluorescence called ‘high nucleic acid’ (HNA) and one with low fluorescence called ‘low nucleic acid’ (Lebaron et al. 2001). For viruses, two populations of VLP could be distinguished based on their fluorescence intensity. HNA could potentially indicate active and fast growing bacteria (Lebaron et al. 2001), while high fluorescence viruses are thought to potentially be algal viruses rather than bacteriophages (Brussaard and Martinez 2008).

**Figure 1.**
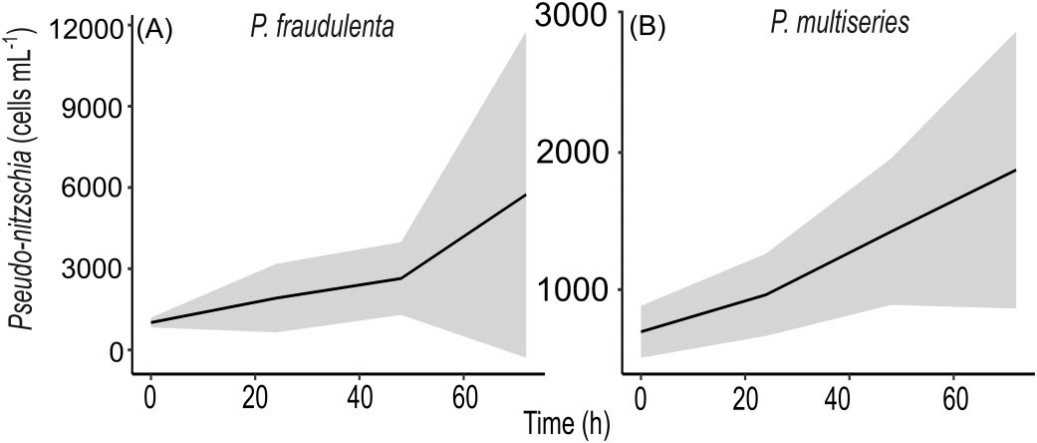
Cell abundance of (A) *Pseudo-nitzschia fraudulenta* and (B) *Pseudo-nitzschia multiseries*. Black lines represent the mean and grey zone the standard deviation of all replicates and levels of turbulence for each time point. Note scale difference.

**Figure 2.**
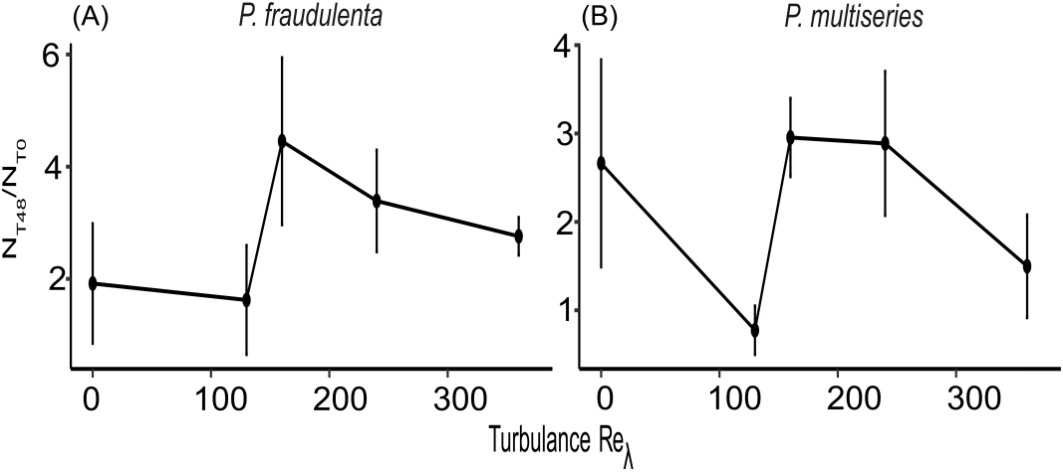
Cell abundance ratio $( {{{\rm {{N}_{T48}}} \!/ \!{{\rm {N}_{T0}}}}} )$ of *Pseudo-nitzschia fraudulenta* (A) and *Pseudo-nitzschia multiseries* (B) versus turbulence intensity. Vertical bars represent the standard deviation of three replicates.

Cell abundance increased during the experiment for both species and in all turbulence conditions, however, standard deviation dramatically increased after 48 h (Fig. 1). Samples of T_48_ were selected for DA measurements, metabarcoding, and metatranscriptomic analyses. These samples were chosen because of the relatively lower standard deviation observed between replicates of the *Pseudo-nitzschia* abundance (Fig. 1) and cell abundance ratio compared to T_72_ (Fig. 2 and Fig. S2), as well as, the fact that, the chains were formed between T24 and T48 h.

For total domoic acid (tDA) measurement 50 ml were sampled in each replicate. Samples were sonicated then acidified with formic acid and concentrated with a solid phase extraction column (Agilent Cartridge Bond Elut C18). The elution was done with a mix methanol/water (50/50 v/v) and kept at −80°C until further analyses. DA measurements were performed using liquid chromatography coupled with mass spectrometry in tandem (LC/MS-MS) as described by Ayache et al. (2019). The cell specific DA calculated here is indicative and was obtained by normalizing the total DA (tDA, in ${\mathrm{pg\ m}}{{\mathrm{l}}}^{ - 1})\ $to the cell density for each sample (${\mathrm{cells\ m}}{{\mathrm{l}}}^{ - 1}$).

For metabarcoding and metatranscriptomics analyses of each tank and at each time point, $200{\mathrm{\ ml}}$ were immediately filtered on sterile filtration devices. To favour the study of the epiphytic bacteria community, $2$ ${\mathrm{\mu m}}$ Nuclepore filters (47 mm, Millipore, USA) were used, permitting as many free-living bacteria as possible to be eluted. Samples were then stored at –80°C until nucleic acids extraction.

The physical linkage between *Pseudo-nitzschia* and the sequenced bacteria was examined using scanning electron microscopy (SEM). A filtration was made on a 2 µm Isopore filters (25 mm, Millipore, USA) to eliminate the glutaraldehyde used for conservation. Then, samples were dehydrated in a graded series of ethanol (50%, 75%, 90%, and 100%) for 30 min at each grade and in a final bath of hexamethyldisilazane. Finally, samples were coated with gold palladium before being observed with a SEM (Hitachi S-3200 N).

### DNA extraction, 16S rRNA gene amplicon sequencing, and processing of sequences

DNA extraction was performed for the T48 samples following the AllPrep DNA/RNA kit (Qiagen, Hilden, Germany) following manufacturer’s protocol. Metabarcoding was used to describe bacterial diversity. 16S rRNA gene amplicon next generation sequencing library preparations and Illumina sequencing were conducted at Azenta Life Sciences (South Plainfield, NJ, USA). Sequencing library was prepared using the MetaVx™ 16S-EZ 16S rRNA gene amplicon library preparation kit (Azenta Life Sciences, South Plainfield, NJ, USA). The selected kit amplifies the V3 and V4 hypervariable genomic regions using the primer set of the 16S-EZ protocol. Indexed adapters were added to the ends of the 16S rRNA gene amplicons by limited cycle PCR. Then, DNA libraries were validated and quantified before loading. The pooled DNA libraries were loaded on an Illumina MiSeq instrument according to manufacturer’s instructions (Illumina, San Diego, CA, USA). The samples were sequenced using a 2 × 250 paired-end configuration.

Demultiplexed 16S gene sequences (i.e. 11 870 156 reads) were processed with the R package DADA2 (Callahan et al. 2016) in order to identify amplicon sequence variants (ASVs). The pipeline includes several steps. First, the primers were removed using the filtering parameters [i.e. maxN = 0, minLen =200, maxEE (5,5), and truncLen (240, 240)]. Then, identical sequences were dereplicated and an abundance was associated with each unique sequence. After, a parametric model was used to learn the error rate for each sequencing run to identify ASVs. Then, forward and reverse sequences were merged. Finally, the chimeric sequences were removed with the removeBimeraDeNovo function (DADA2), using the consensus method. A total of 7 884 165 reads were remaining after these steps, corresponding to 4437 ASVs. Taxonomic annotation was performed with the RDP Naive Bayesian Classifier using the SILVA reference data base (release 138; Wang et al. 2007, Quast et al. 2013). Samples were rarefied at 51 649 sequences (i.e. the lowest number of sequences in a sample) with phyloseq package (v.1.42.0; McMurdie and Holmes 2013). All ASVs sequences were aligned in Geneious Prime (v.2023.0.4) using MUSCLE algorithm (v.5.1 ; Edgar 2004). A phylogenetic tree was then built based on this alignment with FastTree plugin using default parameters (v.2.1.11; Price et al. 2009). ASVs not assigned to procaryotes or assigned to mitochondria and chloroplasts were removed as well as singletons leaving a total of 1999 ASVs and 1 807 715 reads in 35 samples. One replicate sample (*P. multiseries*  ${\mathrm{R}}{{\mathrm{e}}}_{\mathrm{\lambda }} = 160$) was removed due to the low quality of its reads.

Raw sequencing data have been submitted to the Short Read Archive under BioProject ID PRJNA980977.

### Metatranscriptomic analysis

In the case of *P. multiseries*, where previous work about the DA production locus exists (Brunson et al. 2018), RNA extraction was performed at samples at 48 h at R${{\mathrm{e}}}_{\mathrm{\lambda }}$ = 160 and in the ‘still conditions’ (R${{\mathrm{e}}}_{\mathrm{\lambda }}$ = 0), in order to assess the overall system and the DA functional profile shifts under these treatment regimes, as follows: total RNA was extracted from flash-frozen 0.2-µm nucleopore filters containing, using the AllPrep DNA/RNA kit (Qiagen), along with DNA extractions. RNA sequencing was performed by Genewiz with the NEBNext Ultra II RNA Library Preparation Kit (New England Biolabs, Ipswich, MA, USA) for the library preparation and bacterial and eukaryotic rRNA depletion and the S2 chemistry kit at a NovaSeq 6000 instrument (Illumina) for generating 2 × 150 bp reads. The reads were processed according to the SAMSA2 pipeline (Westreich et al. 2018). Within the pipeline context, Trimmomatic v0.36 (Bolger et al. 2014) was used for quality controlling/filtering the sequence reads using a sliding window of four bases of a minimum mean quality of Phred Q values of 15 as a 3′ trimming cut-off and a minimum length of 70 for post-trimming read filtering cutoff value. PEAR v0.9.10 (Zhang et al. 2014) was then used for read-pair assembly with the default parameters. The contigs derived from read-pairs were then screened with SortMeRNA v2.1 (Kopylova et al. 2012) for prokaryotic 16S and 23S rRNA, for eukaryotic 18S and 28S rRNA, and for 5S and 5.8S rRNA gene sequence remnants. The rRNA-free assembled read-pairs were then contrasted with the Diamond v2.0.11 BLASTx algorithm, using the default parameters and retaining the best hit, against the RefSeq and the SEED Subsystems databases as maintained by the SAMSA2 group with the databases further enriched for NCBI residing *Pseudo-nitzschia* associated sequences of the DA associated genes as suggested by Brunson et al. (2018).

### Statistical Analysis

All statistical analyses were performed in R version 4.2.2. Data visualizations were made with the R package ggplot2 (Wilkinson 2016). For bacteria alpha-diversity indices (Observed richness, inverse Simpson, and Shannon) were calculated with the phyloseq package (McMurdie and Holmes 2013).

To illustrate ${\mathrm{\beta }}$-diversity, a nonmetric multidimensional scaling (NMDS) ordination plot was preformed based on weighted UniFrac metric (Lozupone and Knight 2005). With this algorithm, a distance matrix between bacterial communities based on the phylogenetical distances between the sequences of the samples was obtained. The weighted parameter was used to assess a weight of each sequence based on their relative abundance in the sample. A distance-based permutational multivariate analysis of variance (PERMANOVA; Anderson 2001) was used to evaluate the statistical significance of differences between group centroids.

The counts matrix derived from the RNA sequencing data was used for identifying differential expression analysis between the two tested conditions by performing a Fisher’s exact test.

## Results

### Cell abundance, chain formation, Chl *a*, and DA

Concentrations of nitrate (NO_3_^−^), nitrite (NO_2_^−^), phosphate (PO_4_^3−^), and silicate Si(OH)_4_ were measured daily with all indicating decreases during the first 48 h. After 48 h, all nutrients were not depleted, with a minimum concentration for nitrogen of $6.07 \pm 2.94\,{\mathrm{\mu M\ }}$ (Table S1). Cell abundance increased for both strains from the beginning until the end of the experiment (72 h, Fig. 1). The mean abundance of *P. fraudulenta* ranged from 1012 to 5740 cells ml^−1^ and was higher than the one of *P. multiseries*, which ranged from 700 to 1851 cells ml^−1^. An increase in the standard deviations between replicates was observed over time for both strains in all turbulence conditions (Fig. 1). At 48 h the standard deviation between replicates was lower than for 72 h (Figs. 2 and Fig. S2). For both strains, maximum cell abundance at 48 h was found for intermediate turbulence levels (${\mathrm{R}}{{\mathrm{e}}}_{\mathrm{\lambda }} = 160$ or 240, Fig. 2).

**Figure 3.**
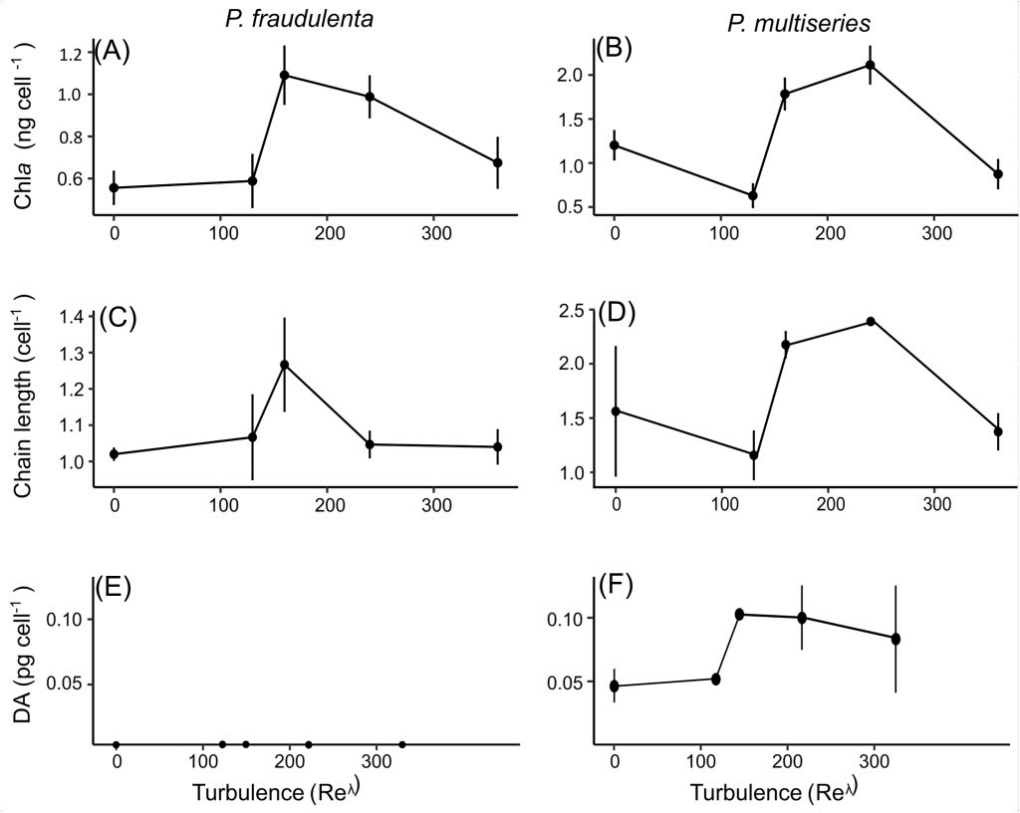
Content in Chl *a* per cell at 48 h of (A and B), mean number of cells in chains (C and D), and DA concentrations and DA normalized per cell at 48 h (E and F). No DA was found in *P. fraudulenta* samples. Vertical bars represent the standard deviations between the different replicates.

Chl *a* concentrations were expressed in µg cell^−1^. At 48 h, maximum Chl *a* per cell was found for intermediate turbulence at ${\mathrm{R}}{{\mathrm{e}}}_{\mathrm{\lambda }} = 160$ and ${\mathrm{R}}{{\mathrm{e}}}_{\mathrm{\lambda }} = 240$ for *P. fraudulenta* and *P. multiseries*, respectively (Fig. 3A and B). To note that, phaeopigments were undetectable in the growing cultures. At the beginning of the experiment, 92% of cells were single with this number dropping to 80% after 72 h of culture. The chains observed at 72 h were relatively short with a mean $1.1 \pm 0.13\ {\mathrm{cell\ chai}}{{\mathrm{n}}}^{ - 1}\ $ for *P. fraudulenta* and $1.7 \pm 0.5\ {\mathrm{cell\ chain}}{{\mathrm{s}}}^{ - 1}$ for *P. multiseries*. At 48 h, maximum chain formation was also found for intermediate turbulence (${\mathrm{R}}{{\mathrm{e}}}_{\mathrm{\lambda }} = 160$ or $240$; Fig. 3C and D).

Free-living heterotrophic bacteria measured with flow cytometry during the experiments ranged from $5.1\ \times {10}^3$ to $7.49\ \times {10}^5\ {\mathrm{cells\ m}}{{\mathrm{l}}}^{ - 1}$. Higher free bacterial abundance was found in *P. multiseries* cultures than in *P. fraudulenta*. Moreover, in both strains, bacteria were more abundant in low turbulence (${\mathrm{R}}{{\mathrm{e}}}_{\mathrm{\lambda }} = 130$; Fig. S3). The mean$\ \pm \ {\mathrm{SD}}$ of all replicates and turbulence conditions of HNA bacteria represented $22.2\% \pm 19.2\%$ of the entire community at the beginning of the experiment and increased linearly, reaching $\ 54.3\% \pm 15.4\% $ after 72 h. Total VLP during this study ranged from $0.01$ to $3.1\ \times {10}^5\ {\mathrm{m}}{{\mathrm{l}}}^{ - 1{\mathrm{\ }}}$($0.2 \pm 0.5\ x\ {10}^5\ {\mathrm{m}}{{\mathrm{l}}}^{ - 1}$). The high fluorescence intensity virus accounted for $8.7\% \pm 6\% $ of all VLP (mean$\pm {\mathrm{SD}}$ of all replicates and turbulence conditions, data not shown).

tDA was measured at 48 h. No toxin was found in *P. fraudulenta*. In *P. multiseries*, toxin was found in all turbulence conditions. Concentrations ranged from $11.1$ to $211.8\ {\mathrm{pg\ m}}{{\mathrm{l}}}^{ - 1}$. These tDA concentrations were normalized relative to the number of cells in each sample. Maximum DA concentrations in the tanks and DA normalized per cell were found for intermediate turbulences (${\mathrm{R}}{{\mathrm{e}}}_{\mathrm{\lambda }} = 160$ or $240$). However, considerable standard deviation between replicates was observed (i.e. in *P. multiseries*, ${\mathrm{R}}{{\mathrm{e}}}_{\mathrm{\lambda }} = 160,$ DA was $0.008 \pm 0.005\ {\mathrm{pg\ cel}}{{\mathrm{l}}}^{ - 1}$, Fig. 3E and F).

### Bacterial community associated with *Pseudo-nitzschia* spp.

Observations of *Pseudo-nitzschia* under the SEM confirmed the presence of epiphytic bacteria on the phytoplankton cells, attached together with mucus and the presence of free-living bacteria remaining on the 2-µm filters (Fig. S4A–D). Sequencing of the 16S rRNA gene showed several differences between bacterial communities associated with *P. multiseries* and *P. fraudulenta*. Out of the total of $1999$ ASVs detected, $1027\ $ were only present in *P. fraudulenta* samples, $517$ were only present in *P. multiseries* samples, and $455$ were shared between both strains (Fig. S5A). Richness was higher in the nontoxic *P. fraudulenta* samples (ranging from 61 to 418 ASVs per sample) than in the toxic *P. multiseries* samples (ranging from 100 to 174 ASVs). Bacterial communities were also more diverse in intermediate turbulences with maximum Simpson and Shannon indices at ${\mathrm{R}}{{\mathrm{e}}}_{\mathrm{\lambda }} = 160.$ (Fig. S5A). The highest variability in alpha diversity between replicates were observed in still and storm conditions (${\mathrm{R}}{{\mathrm{e}}}_{\mathrm{\lambda }} = 0$ and $360\ $; Fig. S5C and D).

**Figure 4.**
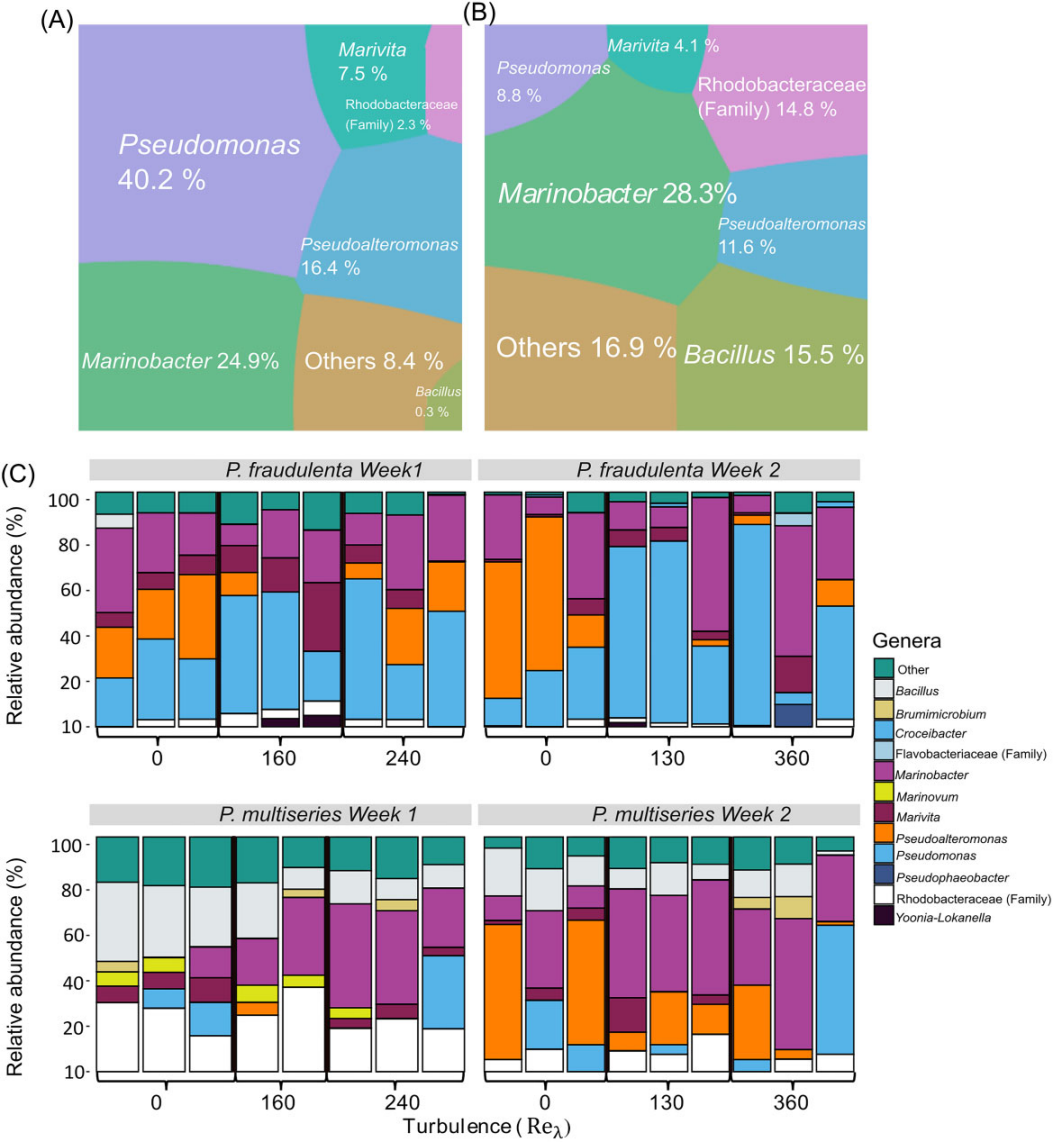
(A and B) Voronoi plot representing the relative abundance, given in percentage of the most abundant bacterial genera in (A) *P. fraudulenta* and in (B) *P. multiseries* cultures at 48 h. Only the genera shared between both cultured strains are represented here. (C) The five most abundant genera of each sample at 48 h for each week of experiment versus turbulence intensity. Each bar represents a replicate. ${\mathrm{R}}{{\mathrm{e}}}_{\mathrm{\lambda }}$=160 for *P. multiseries* only two replicates because of the low the quality reads of one replicate.

In all samples, *Pseudomonas, Pseudoalteromonas*, and *Marinobacter* were the most abundant genera. However, their relative abundances were different. *Pseudomonas* dominated in *P. fraudulenta* cultures, while *Marinobacter* dominated in *P. multiseries* cultures. *Bacillus* (belonging to the phylum Firmicutes) was an important genus in *P. multiseries* (15.5%) but low in *P. fraudulenta* (0.3%; Fig. 4A and B). The abundance of *Bacillus* in *P. multiseries* cultures was higher in the samples with the highest DA concentrations. (Fig. S6).

The relative abundance of the five most abundant genera of each sample was plotted versus the turbulence intensity (Fig. 4C). A high variability was observed between weeks and replicates, and thus, no clear trend could be established between turbulence intensity and bacterial community structure.

**Figure 5.**
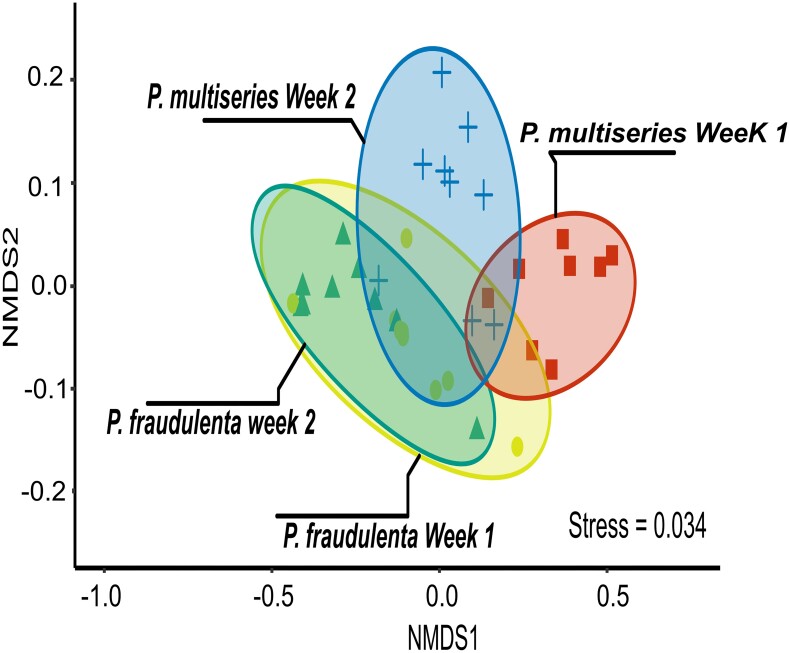
NMDS based on Unifrac Weighted distances for bacteria. All turbulence levels, different colours represent different weeks of experiment, each point represents a sample. treatment.

An NMDS plot indicated significant differences between the bacteria communities associated with *P. fraudulenta* and *P. multiseries* cultures (PERMANOVA, *P* < .05), but did not clearly cluster according to turbulence intensities (Fig. 5). Finally, to investigate the relationship between the level of turbulence and the observed variability between replicates, coefficients of variation of the read abundance were calculated for the 12 most abundant genus at each turbulence intensity. The lowest variability was observed in intermediate turbulence with a mean ${{\mathrm{C}}}_{\mathrm{v}} = 55{\mathrm{\% \ }}$at ${\mathrm{R}}{{\mathrm{e}}}_{\mathrm{\lambda }} = 160$. Maximum variability was found for extreme conditions (Still and Storm) with respectively ${{\mathrm{C}}}_{\mathrm{v}} = 86{\mathrm{\% }}$ and ${{\mathrm{C}}}_{\mathrm{v}} = 109{\mathrm{\% }}$ (Fig. S7).

### mRNA analysis

A total sum of ∼26 M read-pairs were obtained from each sample (i.e. the ‘still condition’; Re_λ_ = 0; 15.6 M read-pairs) and the intermediate turbulence treatment (Re_λ_ = 160; 10.6 M read-pairs) of the *P. multiseries* experimental series. Out of these, ∼ 61% were of high enough quality (after merging, retaining the merged or first reads of each pair for avoiding double-counting, and removal of remaining ribosomal sequences) for the downstream analysis.

Comparison between the Re_λ_ = 160 and the Re_λ_ = 0 of the *P. multiseries* experiment showed that the vast majority of the microbiome functional categories were, on average, downregulated under turbulence conditions, whereas DA biosynthesis and photosynthesis were the only metabolic activities that were significantly upregulated (for cutoff values of 2 log_2_ fold change and *P* < 0.05; Fig. S8A). Out of the four genes of the suggested DA locus (Brunson et al. 2018), *dabA* and *dabC* showed the highest log_2_ fold changes (Fig. S8B), genes that code for a cyclase-like protein and an a-ketoglutarate (aKG)-dependent dioxygenase, respectively (Fig. S8C).

## Discussion

Previous studies dealing with the effect of turbulence on diatom growth used the ‘still condition’ and a single turbulent condition (Clarson et al. 2009, Amato et al. 2017, Dell’aquila et al. 2017). However, the present study investigated, for the first time, the effect of a variable turbulence level (from ${\mathrm{R}}{{\mathrm{e}}}_{\mathrm{\lambda }} = 0$ to ${\mathrm{R}}{{\mathrm{e}}}_{\mathrm{\lambda }} = 360$) on several variables including DA production of two species of the marine diatom *Pseudo-nitzschia* and their associated bacterial communities. The major findings of this work were that the cell abundance, DA production, chain formation, and Chl *a* content of *P. fraudulenta* and *P. multiseries* were higher for intermediate turbulence. This study also highlighted a higher richness of the bacterial community associated with the nontoxic strain of *P. fraudulenta* in comparison to the toxic strain of *P. multiseries*. Furthermore, these bacterial communities did not seem to be directly impacted by turbulence intensity. This absence of clear trend related to turbulence intensity could be also due to the high variability observed: (i) between replicates; (ii) between W1 and W2 for the same strain; and (iii) between strains.

### Effect of turbulence intensity on *Pseudo-nitzschia* spp.

A major constraint when studying *Pseudo-nitzschia* spp. is the inter- and intrastrain variability (e.g. Thessen et al. 2009) and is likely to be one of the many facets illustrating the difficulties encountered when studying planktonic organisms, and in particular *Pseudo-nitzschia*, in laboratory cultures (Shi et al. 2009, Lema et al. 2017). This high variability can explain why there is still today no clear consensus on the conditions triggering the HABs of *Pseudo-nitzschia*.

In our results, high variability was observed between replicates as well as between the 2 weeks of the experiment. Large variability between replicates might derive from the instability of *Pseudo-nitzschia* cells in culture, especially exacerbated by the large volume of culture, despite the attention paid to introduce the same inoculum concentration and the same growth stage, and the same controlled conditions of culture (light, nutrients, and temperature). Indeed, large volumes (15 l) were used to have precise, quantifiable, and homogenous levels of turbulence. The decrease in variability between replicates at intermediate turbulences observed in this study has not been reported before and indicates better growth conditions (Fig. S7). The highest cell abundances were observed for both strains at intermediate turbulence intensities (${\mathrm{R}}{{\mathrm{e}}}_{\mathrm{\lambda }} = 160$ or $240$), while minimum cell abundance was observed in low turbulence levels and still conditions. This is the first evidence that a diatom presented a ‘dome-shape’ response to turbulence as previously described for other marine organisms (i.e. for zooplankton: Cury and Roy 1989, Sundby and Fossum 1990, MacKenzie et al. 1994, and Le Quiniou et al. 2022). The same pattern was found for the chain formation, with the longest chains found at ${\mathrm{R}}{{\mathrm{e}}}_{\mathrm{\lambda }} = 160\ $ for *P. fraudulenta* and ${\mathrm{R}}{{\mathrm{e}}}_{\mathrm{\lambda }} = 240$ for *P. multiseries*. As detailed by Dell’aquila et al. (2017), an increase in chain formation leads to an optimized surface-to-volume ratio and thus increases the probability of the cell to encounter a nutrient-rich zone when placed in a turbulent environment. Typically *Pseudo-nitzschia* forms relatively long chains *in situ* (e.g. Swan and Davidson 2007). The morphological trait of chain formation tends to disappear in culture (Smayda and Boleyn 1966). Two cell chains are described in cultures before (e.g. Amato et al. 2017).

Interestingly, our results confirm that the formation of chains was higher in a turbulent environment than in a still condition in accordance with observations of Wadt et al. (2017), who reported a stimulation of chain formation with constant swirling of cultures. However, the highest level of turbulence used in our experiments (${\mathrm{R}}{{\mathrm{e}}}_{\mathrm{\lambda }} = 360$) had a negative effect on both *Pseudo-nitzschia* strains. For example, the decrease in chain formation observed in storm conditions (${\mathrm{R}}{{\mathrm{e}}}_{\mathrm{\lambda }} = 360$) applied here has not been reported before. Two hypotheses could explain the chain formation decrease: (i) the high levels of turbulence tend to break the chains; (ii) the concentrations of dissolved nutrients become more homogeneous in higher turbulence, leading to a loss of the advantage associated with the chain formation. During the culture of *P. fraudulenta* and *P. multiseries*, an increase of Chl *a* concentration per cell was also observed in intermediate turbulence intensities (${\mathrm{R}}{{\mathrm{e}}}_{\mathrm{\lambda }} = 160$ or ${\mathrm{R}}{{\mathrm{e}}}_{\mathrm{\lambda }} = 240$, respectively). This observation implies that very high turbulence intensity had a negative effect on these diatoms. However, it is important to remember that the experiments were run during two consecutive weeks (W1 and W2) for each strain with three turbulence intensities each week (${\mathrm{R}}{{\mathrm{e}}}_{\mathrm{\lambda }} = 0;160;240$ and ${\mathrm{R}}{{\mathrm{e}}}_{\mathrm{\lambda }} = 0;130;360,\ {\mathrm{repsectively}}$). Furthermore, the average cell size of a diatom population decreases at each cell division during the vegetative phase. The lower Chl *a* content observed in W2 could thus be simply explained by a smaller average cell size (e.g. Jewson 1992) but no size decrease was observed during our study between W1 and W2.

Regarding the production of the toxin DA, it should be noted that unlike *P. multiseries*, which has been shown in numerous studies to be capable of producing DA (e.g. Bates et al. 1991), the toxin production by *P. fraudulenta* is still debated (e.g. toxic for Tatters et al. 2012; and nontoxic for Teng et al. 2016). During our experiments, none of the *P. fraudulenta* samples had detectable levels of DA. This result supports the finding of Dong et al. (2020), that *P. fraudulenta* produced DA only in the presence of grazers. In contrast, all the *P. multiseries* samples exhibited DA concentrations ($73 \pm 58\ {\mathrm{pg\ m}}{{\mathrm{l}}}^{ - 1}$) in the range of the highest values found in the open ocean. On a transect in the Eastern Atlantic Ocean, Geuer et al. (2019) found a maximum concentration of dissolved DA of $53.9{\mathrm{\ pg\ m}}{{\mathrm{l}}}^{ - 1}$ with a mean of $9.9\ {\mathrm{pg\ m}}{{\mathrm{l}}}^{ - 1}$ in the water column. It has been also highlighted that DA production was reduced in culture conditions compared to the production in the natural environment. Depending on the *P. multiseries* strain studied, DA content can vary from $3\ \times {10}^{ - 4}$ to $4,8\ {\mathrm{pg\ cel}}{{\mathrm{l}}}^{ - 1}$ (Dong et al. 2020). In our experiments, the highest DA concentration normalized per cell number was $0.14\ {\mathrm{pg\ cel}}{{\mathrm{l}}}^{ - 1}$.

The biosynthetic pathway of DA involves four identified genes (Brunson et al. 2018). The occurrence (but not its expression) of the *dabA* gene has been recently reported in two (*P. multistriata* and *P. delicatissima*) out of five cultures of *Pseudo-nitzschia* species isolated from the Adriatic Sea (Turk Dermastia et al. 2022), but the production of DA was detected only in *P. multistriata*. In our study, DA synthesis was further confirmed by metatranscriptomics analysis of selected samples from the same experiments where four of the known DA biosynthesis genes were highly expressed after 48 h from the beginning of the experiment at Re_λ_ = 130 compared to the control in the *P. multiseries* tanks (Fig. S8). According to recent research, the induction of DA biosynthesis was associated, in a species-specific manner, with growth phase (Sauvey et al. 2023), and with photosynthetic conditions (Brunson et al. 2018). Our results collectively suggest that the turbulence (an abiotic stress) related induction of the expression of the *P. multiseries* DA production locus is linked to an increase of photosynthetic activity in the total metatranscriptome. DA production has been previously deemed energy demanding (LeLong et al. 2012), and may possibly drive the observed upregulation of the photosynthetic genes.

### Relationship between *Pseudo-nitzschia* spp. and the associated bacteria under a gradient of turbulence

The highest concentration of free-living bacteria measured was $7.49 \times {10}^5\ {\mathrm{cells\ m}}{{\mathrm{l}}}^{ - 1}$. This is in the same range of the mean concentration of prokaryotic cells in the upper 200 m of the open ocean (e.g. $5 \times {10}^5{\mathrm{\ cells}}\ {\mathrm{m}}{{\mathrm{l}}}^{ - 1}$). The highest VLP concentration found during the experiment was $3.1 \times {10}^5\ {\mathrm{m}}{{\mathrm{l}}}^{ - 1}$, which was two orders of magnitude lower than what can normally be observed in a coastal environment. As a reference, in the English Channel, VLP concentration ranged from $0.1$ to $5.8 \times {10}^7\,{\mathrm{m}}{{\mathrm{l}}}^{ - 1}$ ($1.6 \pm 0.9 \times {10}^7{\mathrm{m}}{{\mathrm{l}}}^{ - 1}$, 269 from samples collected across all seasons, 2018–2020; Christaki, unpublished data). These results suggest that the importance of free-living bacteria and viruses in the cultures remained minor compared to diatoms. Viruses will not be further discussed here.

The highest abundance of free-living bacteria in samples of both strains were observed in low turbulence conditions (${\mathrm{R}}{{\mathrm{e}}}_{\mathrm{\lambda }} = 130$; Fig. S3). Some studies have already showed a higher bacterial growth and activity in turbulent conditions (e.g. Bergstedt et al. 2004). Due to their small size ranging from $0.2\ $to $1{\mathrm{\ \mu m}}$, heterotrophic bacteria are smaller than the Kolmogorov and the Batchelor scale, which corresponds to the smallest heterogeneity produce by turbulence. Thus, bacteria should not be affected by turbulence intensity. However, due to their motility, some bacteria are able to navigate through their environment and exploit small ephemeral patches of higher nutrient concentrations (Taylor and Stocker 2012).

In this study, we focused on the epiphytic bacteria by sequencing the 16S rRNA gene of samples filtered on $2{\mathrm{\ \mu m\ filters}}$ to remove most fraction of the free-living bacteria (diameter $\approx 0.5\ {\mathrm{\mu m}}$). However, as it can be seen on the images of diatom under the SEM, some free-living bacteria remained on the filter (Fig. S4). Additionally, the analysis of SSU gene sequences showed that the nontoxic strain of *P. fraudulenta* presented more ASVs (1482) compared to the toxic strain of *P. multiseries* (972). This supports the observation made by Sison-Mangus et al. (2014) that when an algae produces toxin, the diversity of bacterial communities associated with it decreases. This may be because the associated bacteria need to evolve in a host-specific way to resist or even benefit from the toxin and/or that nontoxic algae can interact with a more diverse range of bacteria. Such specific relationships of phycopshere bacteria have been reported for toxic and nontoxic dinoflagellates and remain to be elucidated for *Pseudo-nitzschia* spp. (Deng et al. 2023).

The same pattern was observed for all alpha diversity indices (Richness, Shannon, and Simpson), converging to an increase in diversity at intermediate turbulence (${\mathrm{R}}{{\mathrm{e}}}_{\mathrm{\lambda }} = 130$ or $160$; Fig. S5). The variation of alpha diversity of bacteria associated to phytoplankton relative to turbulence levels has not been assessed until now. While it is tempting to attribute this pattern to the possibility for bacteria with lower or higher turbulence preference to thrive in those intermediate turbulence conditions, this hypothesis remains to be verified.

Important variability in bacterial community structure was observed between replicates, however, this variability was higher for extreme conditions (‘still’ or ‘storm’; Fig. S7). The variability between samples is discussed below. The samples were dominated by the phylum *Proteobacteria*, as it is usually the case in phytoplankton cultures (e.g. Kahla et al. 2021). Depending on the cultivated *Pseudo-nitzschia* strains, different genera were dominant (Fig. 4A and B). The genus *Pseudomonas* was found to be dominant in *P. fraudulenta* samples (40.2% of the whole bacterial community). This genus is known for its ability to occupy a wide range of habitats in water as in soils but has been reported as being inhibited by low levels of DA (Stewart et al. 1998). This sensitivity could explain why *Pseudomonas* represented only 8.8% of the bacterial community associated with *P. multiseries*. In *P. multiseries* samples, the dominant genus was *Marinobacter*, a marine bacteria commonly dominant in the phycosphere (Lupette et al. 2016). None of these taxa appeared to have relative abundances related with the turbulence intensity (Fig. 4C). The lack of a clear effect on the different genus can be explained by the focus made here on the epiphytic community. Attached bacteria probably respond to the diatom variability rather than to the surrounding turbulence intensity. In addition, a small fraction of free-living bacteria remained on the $2\ {\mathrm{\mu m}}$ filters, which has probably amplified the variability in our results (Fig. S4D). This high variability can be clearly observed on the relative abundances of dominant genus in the replicates (Fig. 4C) and is also reflected on the NMDS plot where bacterial communities were grouped mainly by strain rather than by turbulence level (Fig. 5).

The genus *Bacillus* found in *P. multiseries* and *P. fraudulenta* cultures, was the only representative of the phylum Firmicutes. Keeping in mind that taxonomic identification at the species level based on short sequences remains highly uncertain, it is worth noting that 88% of the sequences affiliated to the genus *Bacillus* were assigned to *B. horikoshii*. This bacterium is one of the endosymbiotic bacteria known for its production of a toxin used by their host: the tetrodotoxin (TTX; e.g*. Tetrodontidae*: puffer fish; Lu and Yi 2009). Interestingly, in our results, the relative abundance of *B. horikoshii* was 12 times higher in the toxic cultures of *P. multiseries* than in the nontoxic cultures of *P. fraudulenta*. The relation observed between the relative abundance of *Bacillus* and the concentration of DA normalized to the number of prokaryotic cells is also intriguing and has not been reported before (Fig. S6). The hypothesis that the potential toxic nature of *Bacillus* helps it to adapt its own metabolism to withstand its own toxin, resulting in its resistance to other toxins, as DA produced by *P. multiseries*, could be advanced here. This adaptation could also explain its dominance in toxic environments that are harmful to more sensitive taxa. However, TTX was not measured here, which limits any further interpretation and/or hypothesis around the relationship between *P. multiseries* and *Bacillus*. Besides the potential importance of TTX, *Bacillus* is known to produce extracellular algicidal compounds against phytoplankton such as cyanobacteria, dinoflagellates and haptophytes of the genus *Phaeocystis* (e.g. Mayali and Azam 2004, Guan et al. 2014, Shao et al. 2022). Variation in bacterial communities may affect DA production by *Pseudo-nitzschia* (Lelong et al. 2014). To better characterize the relationship between *P. multiseries* and *Bacillus*, it would be interesting to test the effect of TTX and the effect of *Bacillus* algicidal compounds on toxic *Pseudo-nitzschia* strains, and the effects of DA on the cultures of *Bacillus*.

## Conclusions

Despite the difficulties associated with studying the diatom *Pseudo-nitzschia* spp., general trends are beginning to emerge after several decades of research on this genus by the scientific community. This work contributes to this ongoing effort to understand the ecology of *Pseudo-nitzschia* spp., by incorporating a novel and significant aspect—the effect of turbulence. Indeed, this study provided the first evidence that the cell abundance of *Pseudo-nitzschia*, its tendency to form chains, its pigment content, and its toxin production, were higher at intermediate turbulence levels. This is the first evidence that a diatom presented a ‘dome-shape’ response to turbulence intensities as previously advocated for zooplankton. In order to better understand mechanisms employed by *Pseudo-nitzschia* spp. to adapt to the turbulence of its environment, future studies should focus in the collection and analysis of targeted metatranscirptomic data related to the expression and regulation of DA biosynthesis. Our results point towards the significance of abiotic stresses on DA production with photosynthetic pathways going hand-in-hand in this phenomenon. The bacterial communities associated with *Pseudo-nitzschia* spp. have also been studied, with a focus on epiphytic bacteria. Corroborating with precedent research, toxic strains have a less diverse associated bacterial community compared to nontoxic strains. Furthermore, the study of associated bacteria has highlighted the genus *Bacillus* (and potentially the species *B. horikoshii*) as being closely associated with the concentrations of DA produced by *P. multiseries*. This relationship warrants further in-depth investigation to better understand the role of the metabolites produced by one species that either promote or restrict the growth of the other. The major difficulty in this study was the high levels of variations observed in the cell abundance of *Pseudo-nitzschia* as well as in the associated bacterial communities. However, this variability appears to be reducible by a better understanding of the optimum growth conditions of the strains studied. One of the effects of turbulence, which is not taken into account in this study but cannot be neglected in the objective of modelling and predicting HAB events, is the vertical mixing on decameter-scale depth. Indeed, this mixing will result in the resurfacing of cells adapted to deeper environments (darker and colder) and vice versa. Consequently, these cells would be subjected to stressful conditions more frequently than cells adapted to less turbulent environments (Falkowski 1983).

## Supplementary Material

fiae094_Supplemental_File
