# Supplementary material for: Effects of turbulence on diatoms of the genus Pseudo-nitzschia spp. and associated bacteria
Source: FEMS Microbiol Ecol. 2024 Jul 10;100(8):fiae094. doi: 10.1093/femsec/fiae094 (PMC11264304; doi:10.1093/femsec/fiae094)
Supplement: fiae094_Supplemental_File [file fiae094_supplemental_file.pdf]

Supplementary Table 1. Nutrient concentrations measured during the experiments. Mean ± sd of all replicates and levels of turbulence for each time point.

Samples	NOx (µM)	PO_4(µM)	$Si(OH)_4$(µM)
P. fraudulenta			
T0	22.45 ± 4.35	14.39 ± 1.94	42.40 ± 7.69
T24	14.62 ± 8.6	9.01 ± 2.68	17.45 ± 12.41
T48	6.07 ± 2.94	6.84 ± 1.75	7.29 ± 7.66
T72	7.78 ± 5.34	6.82 ± 1.43	3.44 ± 3.62
P. multiseries			
T0	26.41 ± 5.15	16.06 ± 0.98	46.92 ± 1.29
T24	20.18 ± 13.76	15.05 ± 2.27	46.74 ± 1.34
T48	16.45 ± 11.06	19.26 ± 7.76	22.31 ± 12.09
T72	15.56 ± 7.97	16.43 ± 8.02	20.94 ± 13.1

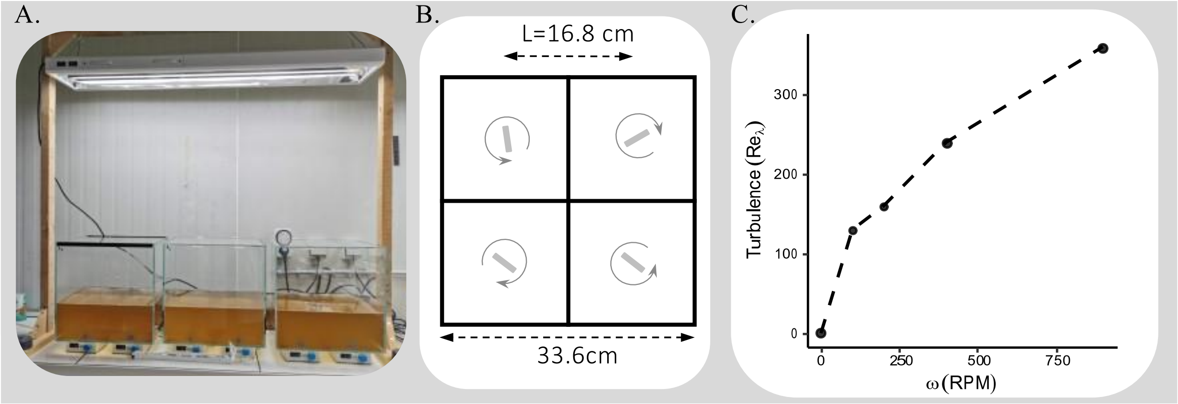

Supplementary Figure 1. Graphical abstract of the experimental set-up. (A) A triplicate Agiturb system during the experiment. (B) Sketch of the experimental setup (top view), L corresponds to the injection scale. (C) Levels of turbulence used during the experiment (Re_λ) versus the speed of revolution (ω) of the agitators in rounds per minute (RPM).

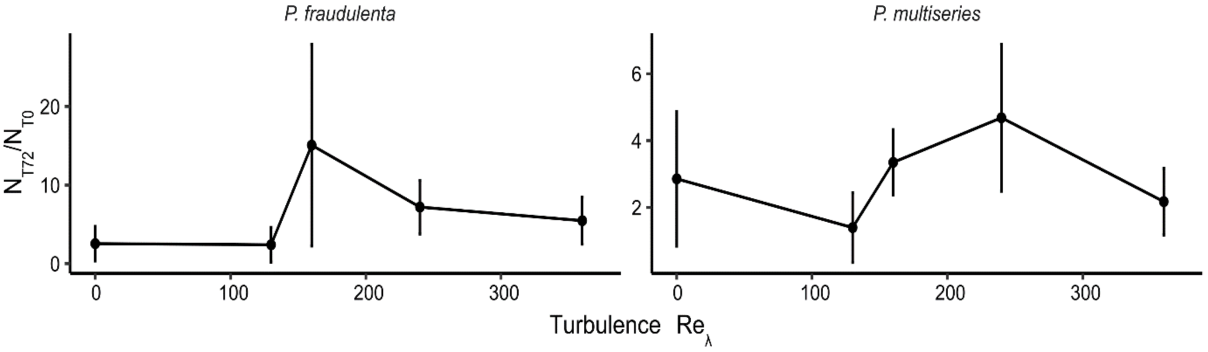

Supplementary Figure 2. Cell abundance ratio $\left({}^{N_{T72}}/_{N_{T0}}\right)$ of both Pseudo-nitzschia strains versus turbulence intensity. Vertical bars represent the standard deviation of 3 replicates.

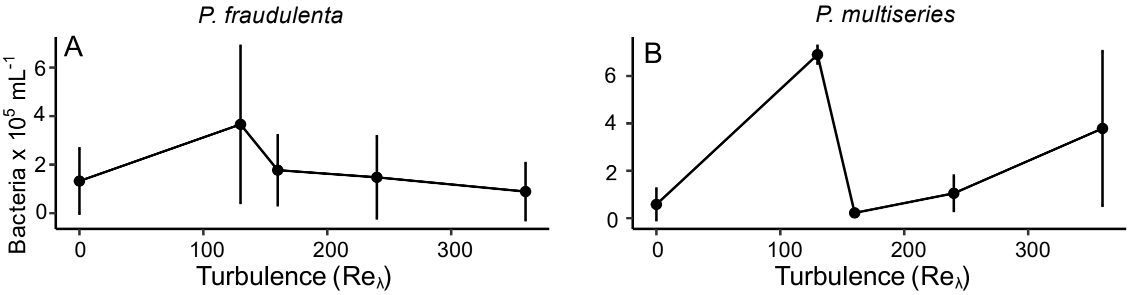

Supplementary Figure 3. Free bacterial cell abundances in the samples at 48h measured with flow cytometry (see M+M)

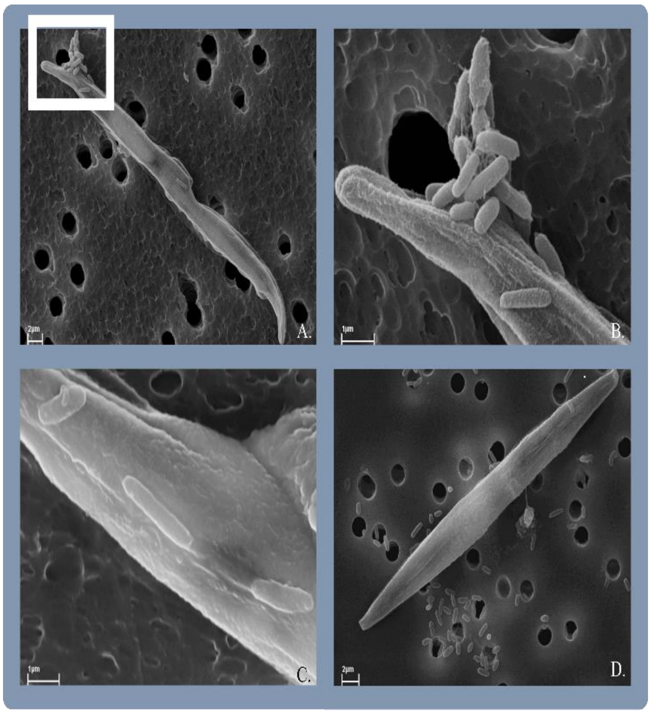

Supplementary Figure 4. Scanning electron microscopy. (A) Chain of *Pseudo-nitzschia fraudulenta* composed of 2 cells. (B-C) Enlargement of the first image with a focus on the epiphytic bacteria. (D) Cell of *Pseudo-nitzschia multiseries* with free-living bacteria. The processing of the samples used to preserve the prokaryotic community does not allow the observation of the ornamentation on the surface of the diatom cell. Linear dimensions of *P. fraudulenta* and *P. multiseries* were: Length , 45.8± 8.1 and 37.3±4.4 µm, respectively and Width, 3.7 ± 0.4 and 3.0 ± 0.4 respectively.

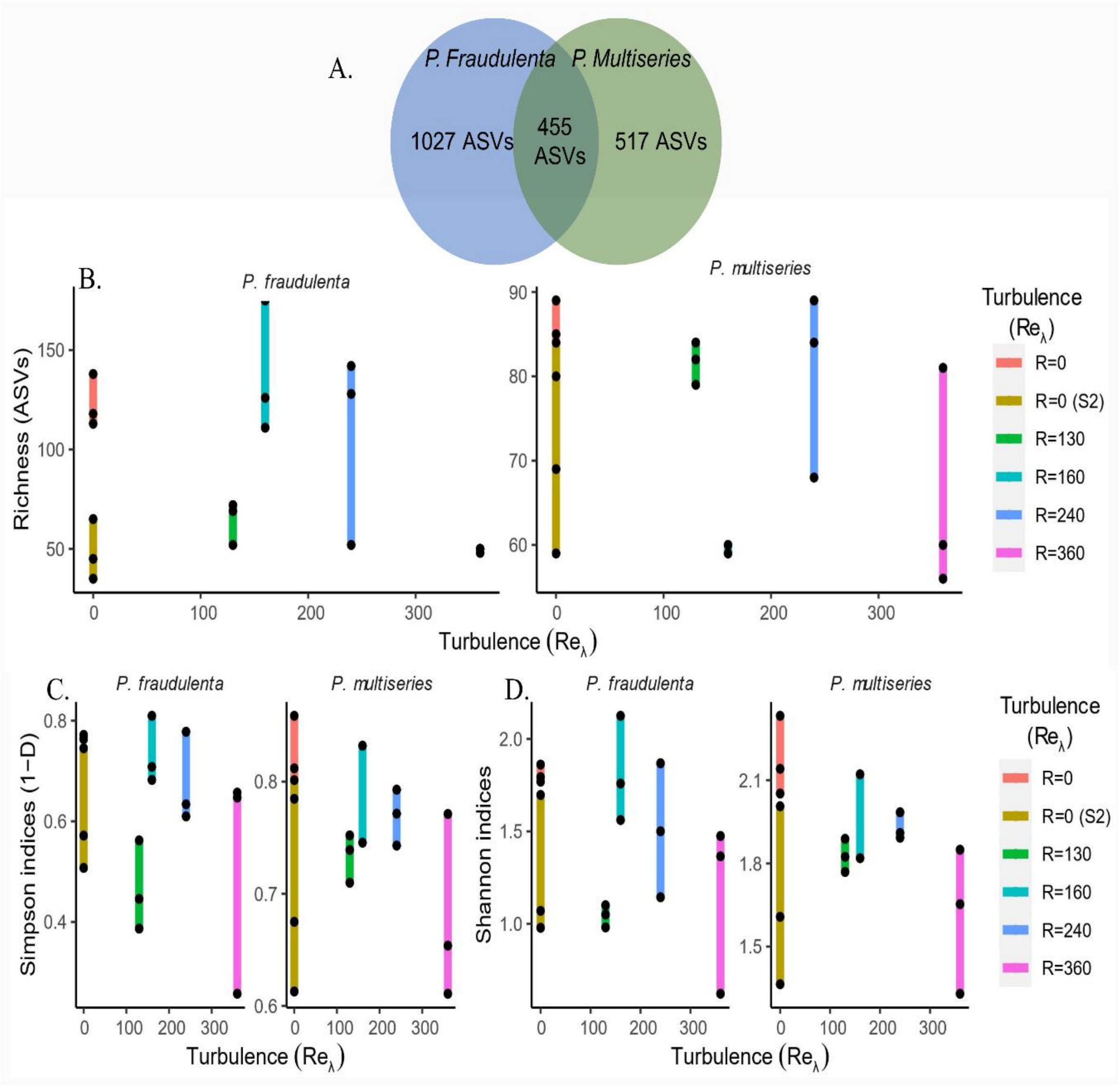

Supplementary Figure 5. Alpha diversity of the bacterial communities based on metabarcoding data. (A) Number of unique and shared ASVs. (B) Richness, each point represents a sample. (C) Simpson and (D) Shannon indices. Vertical colored lines represent the different turbulence intensities.

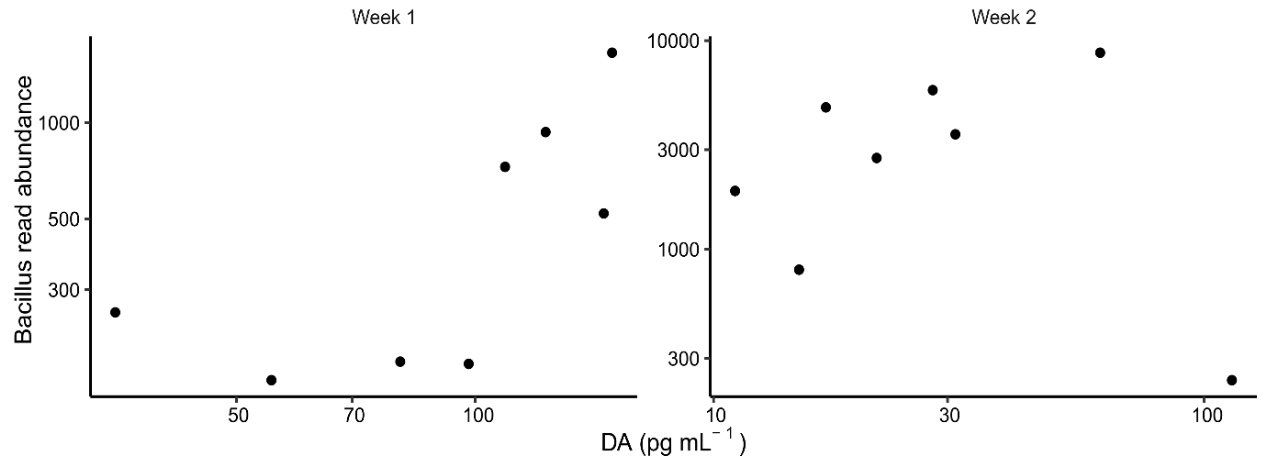

Supplementary Figure 6. Number of reads (i.e., metabarcoding) of the genus *Bacillus* versus DA concentration in *P. multiseries* samples. Note the logarithmic scale on x and y axis.

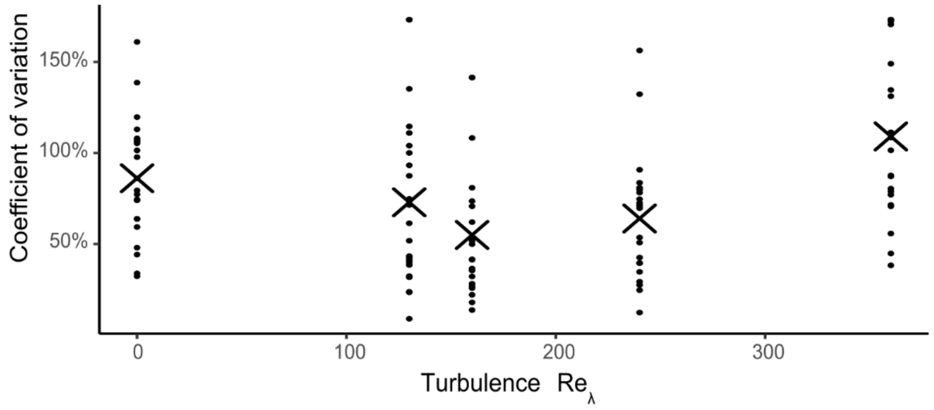

Supplementary Figure 7. Coefficients of variation (C_v) of the abundance of 12 most abundant genera in all replicates. Each point represents the C_v of a genus. Black crosses represent the mean.

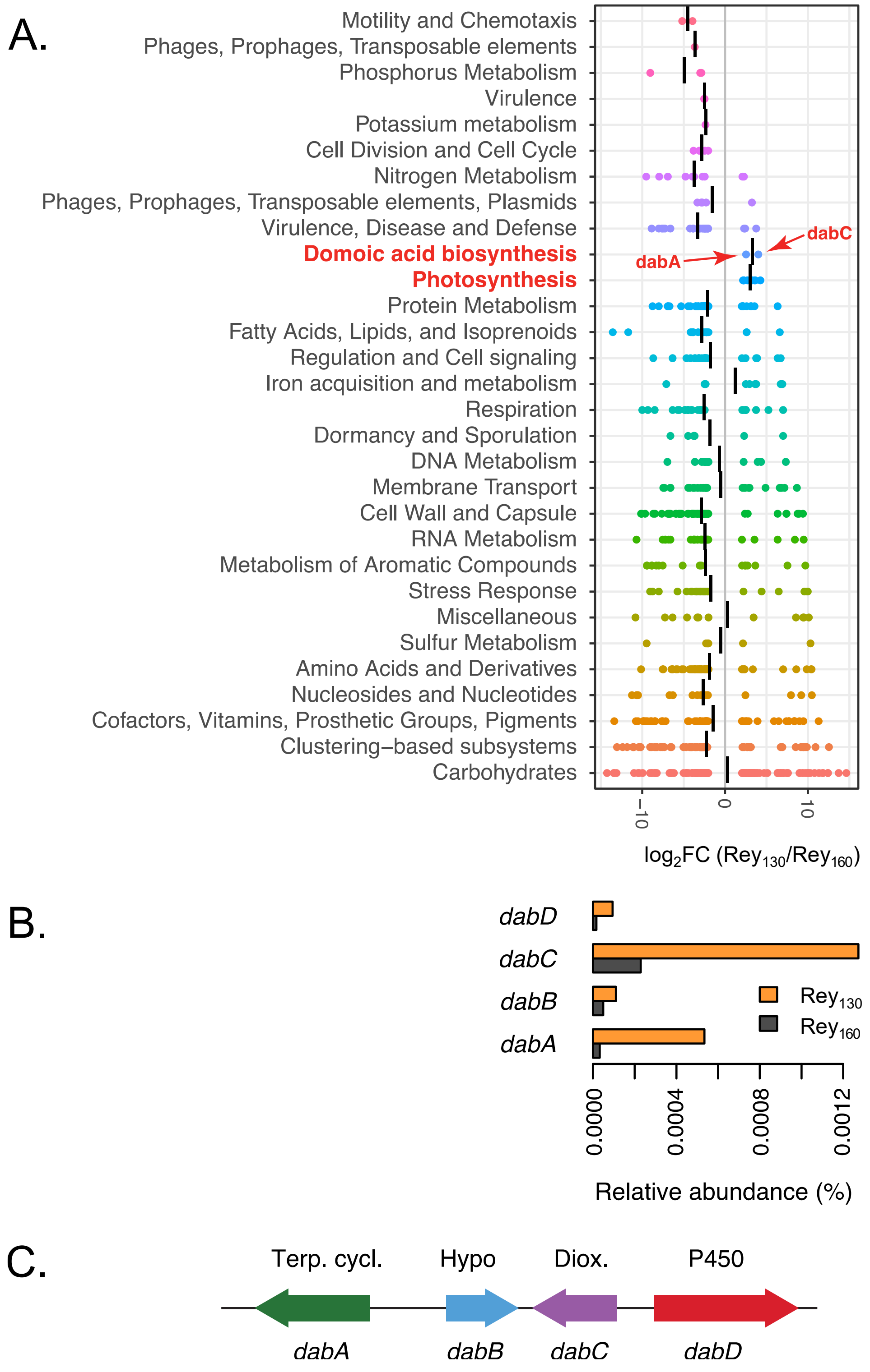

Supplementary Figure 8 (A) $Log_2(Rey_{160}/Rey_0)$ values for statistically significant SEED feature differences according to the Fisher's exact test (α 0.01 and $log_2(Rey_{160}/Rey_0)$ cut-off of 2). Mean $log_2(Rey_{160}/Rey_0)$ values are provided by the black lines for each feature category, with the *P. multiseries* domoic acid biosynthesis genes and photosynthetic annotations being the only ones with increased expression of all their features at the Rey_{160}
